# Quantitative Trait Loci for Salinity Tolerance Identified under Drained and Waterlogged Conditions and Their Association with Flowering Time in Barley (*Hordeum vulgare*. L)

**DOI:** 10.1371/journal.pone.0134822

**Published:** 2015-08-06

**Authors:** Yanling Ma, Sergey Shabala, Chengdao Li, Chunji Liu, Wenying Zhang, Meixue Zhou

**Affiliations:** 1 Tasmanian Institute of Agriculture and School of Land and Food, University of Tasmania, P.O. Box 46, Kings Meadows, TAS, 7249, Australia; 2 Western Barley Genetics Alliance, Murdoch University, 90 South Street, Murdoch, WA, 6150, Australia; 3 CSIRO Plant Industry, 306 Carmody Road, St. Lucia, QLD, 4067, Australia; 4 School of Agriculture, Yangtze University, Jingzhou, 434025, P.R. China; Zhejiang University, CHINA

## Abstract

**Introduction:**

Salinity is one of the major abiotic stresses affecting crop production via adverse effects of osmotic stress, specific ion toxicity, and stress-related nutritional disorders. Detrimental effects of salinity are also often exacerbated by low oxygen availability when plants are grown under waterlogged conditions. Developing salinity-tolerant varieties is critical to overcome these problems, and molecular marker assisted selection can make breeding programs more effective.

**Methods:**

In this study, a double haploid (DH) population consisting of 175 lines, derived from a cross between a Chinese barley variety Yangsimai 1 (YSM1) and an Australian malting barley variety Gairdner, was used to construct a high density molecular map which contained more than 8,000 Diversity Arrays Technology (DArT) markers and *single nucleotide polymorphism (SNP) markers*. Salinity tolerance of parental and DH lines was evaluated under drained (SalinityD) and waterlogged (SalinityW) conditions at two different sowing times.

**Results:**

Three quantitative trait loci (QTL) located on chromosome 1H, single QTL located on chromosomes 1H, 2H, 4H, 5H and 7H, were identified to be responsible for salinity tolerance under different environments. Waterlogging stress, daylight length and temperature showed significant effects on barley salinity tolerance. The QTL for salinity tolerance mapped on chromosomes 4H and 7H, QSlwd.YG.4H, QSlwd.YG.7H and QSlww.YG.7H were only identified in winter trials, while the QTL on chromosome 2H QSlsd.YG.2H and QSlsw.YG.2H were only detected in summer trials. Genes associated with flowering time were found to pose significant effects on the salinity QTL mapped on chromosomes 2H and 5H in summer trials. Given the fact that the QTL for salinity tolerance QSlsd.YG.1H and QSlww.YG.1H-1 reported here have never been considered in the literature, this warrants further investigation and evaluation for suitability to be used in breeding programs.

## Introduction

Salinity stress is one of the major abiotic stresses affecting crop production and many saline soils are also prone to waterlogging, with the resulting root hypoxia reducing growth of dryland cereals [1,2]. The main adverse effects caused by salinity stress can be divided into two major categories 1) osmotic effects and 2) ion toxicity (Na^+^ and Cl^-^) [3]. The osmotic stress can pose immediate effects on plant growth by limiting availability of water to plants [4]. Specific ion toxicity in the shoot takes a longer time to impact plant growth (days or weeks), and shows less effect than the osmotic stress, especially at low to moderate salinity levels [3,4], although in roots NaCl-specific programmed cell death is observed within hours afters salinity stress onset [5]. Yet, the main site of Na^+^ toxicity for most plants is the leaf blade, where Na^+^ accumulates, after being deposited in the transpiration stream, rather than in the roots [3,6]. The cytosolic K^+^/Na^+^ ratio has been repeatedly named as a key determinant of plant salt tolerance [4,7–10]. The optimal cytosolic K^+^/Na^+^ ratio can be maintained by either restricting Na^+^ accumulation in plant tissues or by preventing K^+^ loss from the cell [9].

Barley has a relatively better salt-tolerance among cereal crops and can tolerate a high concentration of Na^+^ in leaf blades [3]. Salinity tolerance in barley is a complex trait controlled by multiple genes [11]. There have been many reports on QTL related with salt tolerance which has been evaluated by numerous traits at the whole plant level. These include yield and agronomic traits, leaf chlorosis, plant survival, shoot sodium content and Na/K ratio (NAK) [11–14]. A single major QTL for salinity tolerance was identified from a Chinese landrace. This QTL was located on chromosome 2H explaining nearly 50% of the phenotypic variation [2]. *HvNax3* and *HvNax4*, conferring the mechanism of sodium exclusion, were found in the short arm of chromosome 7H and the long arm of chromosome 1H, respectively [15,16]. *HvNax4* has been proved as a barley homologue of the *SOS3* salinity tolerance gene of *Arabidopsis* [15]. The *HvHKT1* gene conferring Na^+^ uniport in barley roots has been cloned [11].

Excess water in the root zone of land plants is detrimental or lethal when it forms a barrier between soil and air free transfer of gases, such as O_2_ and CO_2_ [17], with the effect of inadequate oxygen supply being most significant. In addition to the elemental toxicities to the sensitive root tips caused by O_2_ deficiency, increased concentration of secondary metabolites such as phenolics and volatiles fatty acid could become injurious in the rhizosphere [18,19]. Waterlogging stress is often comorbid with salinity. Over-accumulation of Na^+^ and Cl^-^ in shoots under waterlogged conditions is greater than for salinity alone since energy (ATP) stress caused by waterlogging-induced anoxic stress dampened the exclusion of Na^+^ and Cl^-^ [20]. The active transport of Na^+^ out of the plant mediated by plasma membrane Na^+^/H^+^ antiporters [21,22] is suppressed when activity of the plasma membrane H^+^-ATPase is reduced under an anoxia environment [4]. When salinity and root hypoxia occur together, K^+^ export channels may be activated by ROS produced under stress conditions [23–25], leading to a more severe reduction in the uptake of K^+^ [26] as well greatly increased leakage of K^+^ from plant roots.

Flowering time in plants (or heading date in crops), an important trait for plant development, has also been reported to be associated with stress tolerance [27–29]. The interplay between environmental stressors and flowering time has been investigated by plant transcription factors (TFs) at the genetic or epigenetic level and through hormonal interaction [28,30,31]. However, no direct evidence from the perspective of QTL mapping or gene exploration has been provided yet.

In this study, 175 double haploid (DH) lines from a cross between YSM1 and Gairdner were genotyped with Diversity Arrays Technology (DArT), which ended up with more than 5,800 single nucleotide polymorphism (SNP) and 13,500 DArT markers. About 8,500 markers with less distortion and missing data were selected to construct a genetic map. Salinity tolerance was screened under various environments, i.e. summer trial and winter trial, and well-drained or waterlogged saline potting mixture. QTL were mapped for salinity tolerance based on plant survival as described in previous studies [2,14].

## Materials and Methods

### Plant materials

A double haploid (DH) population consisting of 175 lines, derived from a cross between Yangsimai 1 (YSM1) and Gairdner was used to identify QTL conferring salinity tolerance. YSM1 is originated from China with medium tolerance to both salinity [32] and waterlogging stresses (unpublished data). Gairdner is an Australian cultivar and highly-sensitive to salt stress [33]. However, Gairdner also showed medium tolerance to waterlogging stress, compared with two other well-known waterlogging-intolerant cultivars Franklin and Naso Nijo [34]. In addition, four other genotypes, Baudin, Franklin, TAM407227 and Naso Nijo, were included as controls.

### Evaluation of salinity tolerance under drained and waterlogged environments

Both summer and winter trials for salinity tolerance screening were conducted in glasshouses located at Mt Pleasant Laboratories, Launceston, Tasmania. Two treatments were used: 200 mM NaCl with drained condition (SalinityD) as described before [14] and waterlogged (SalinityW) with 200 mM NaCl solution. In the summer trials (from February to April in Year 2014), two replicates were included for each treatment. Eight 40-L containers (each including 18 lines) filled with commercial potting mixture were used for each replicate. All replicates were arranged in a randomized block design. Glasshouse settings for plant growth were 25/15 (±5) °C for day/night temperature under natural daylight. The SalinityD and SalinityW treatments were started at the three-leaf stage, and lasted four weeks. The drainage system and application system connected between containers and pumps for salinity treatment were as previously described [14]. The system approached a steady state where, after 4–5 watering cycles, NaCl additions were minimal and only water was added to replace evaporation and transpiration [14]. For SalinityW treatment the saline solution (200 mM NaCl) was kept in the containers rather than being drained (just above the surface). Winter trials were conducted between June and August in Year 2014. Glasshouse settings for plant growth were 15 /10 (±5) °C for day/night temperature under natural daylight. All treatments were the same as summer trials. The control experiment was not conducted since it has been proved that different varieties or DH lines grown in the same potting mixture but with no salt added showed no apparent symptoms of leaf chlorosis or wilting [2,14]. Salinity tolerance was assessed by combining scores for leaf chlorosis and plant survival two weeks after SalinityW treatment for summer trials and three weeks after SalinityW treatment for winter trials, four weeks after SalinityD treatment for summer trial and five weeks after SalinityD treatment for winter trial (0 = no damage and 10 = all dead).The different scoring times were selected at the stage when significant differences were apparent among parental and control varieties and DH lines.

### Assessment of plant development stages (flowering time)

Plant development stages (flowering time) was scored only in summer trial after 3-week salinity and SalinityW treatments with score ‘1’ = staying at tillering stage with a Zadoks score of less than 29, ‘2’ = early booting stage with a Zadoks score of around 41 and ‘3’ = anthesis stage with a Zadoks score of 65 [35].

### Leaf Na^+^ and K^+^ contents measurement

In summer trials, after three-week SalinityD and SalinityW treatments, the youngest fully expanded leaf was collected and immediately stored in a 1.5 ml microcentrifuge tubes at −20°C. The sap from leaves was extracted by the freeze-thaw method [36]. After centrifuged at 10,000 g for 3 min, the extracted sap sample was diluted 50 times with double distilled water and analysed for Na and K contents using a flame photometer (PF97, VWR International, Murarrie, Australia). Na/K ratio was then calculated. Four replicates of measurement were taken for every leaf sample [37].

### Map construction

Genomic DNA was extracted from the leaf tissue of three-week old seedlings, based on a modified CTAB method described by Stein *et al*. [38]. DH lines and two parental varieties were genotyped with DArTSeq (http://www.diversityarrays.com/dart-application-dartseq). Due to the large number of DNA markers (>5,800 SNP and >13,500 DArTSeq markers), markers with greater distortion and missing data were removed from map construction. A total of 8,528 markers were selected to construct the genetic map. The software package JoinMap 4.0 [39] was used to construct the linkage map.

### QTL and statistical analysis

The average values from each experiment were used for the identification of QTL associated with salt tolerance. The software package MapQTL 6.0 [40] was used to detect QTLs which were first analysed by interval mapping (IM). The closest marker at each putative QTL identified using interval mapping was selected as a cofactor and the selected markers were used as genetic background controls in the approximate multiple QTL model (MQM). A logarithm of the odds (LOD) threshold value of 3.0 was applied to declare the presence of a QTL at 95% significance level. To determine the effects of other traits on the QTL for salinity tolerance, QTL for salinity tolerance were re-analysed by using flowering time and leaf Na/K ratio as covariates. The percentage of variance explained by each QTL (R^2^) was obtained using restricted MQM mapping implemented with MapQTL6.0. Graphical representation of linkage groups and QTL was carried out using MapChart 2.2 [41]. All other statistical analyses, for example, calculation of mean values and standard errors and analysis of variances (ANOVA), were performed using SPSS software package (Version 20.0, IBM).

## Results

### Salinity tolerance of parental varieties and DH lines

After one week of treatment the lower leaves of susceptible genotypes started to wilt. In SalinityW treatment, significant differences in leaf yellowing between the two parental varieties became obvious one-week (summer trials) or two-week (winter trials) post treatment. SalinityW stress caused much greater damage than SalinityD stress at the same time period (Table 1).

**Table 1:** Score values of SalinityD, and SalinityW tolerance and flowering time for two parental varieties and four control varieties in summer trials.

Variety	2-week treatment		3-week treatment		4- week treatment		Flowering time
	SalinityD	SalinityW	SalinityD	SalinityW	SalinityD	SalinityW	
**Gairdner**	6	7.5	7	7.75	6	7.5	3
**YSM1**	3	3	3	3.25	3.5	4	2.25
**Baudin**	3.25	6.25	3.25	6.25	5.5	5.5	3
**TAM704227**	2.25	2	1.25	2	1	0.5	1
**TX9425**	2.5	6	3.5	6.25	5.5	4	2
**Naso Nijo**	5.75	8.75	7	9	8.5	9	2.83

**Fig 1:**
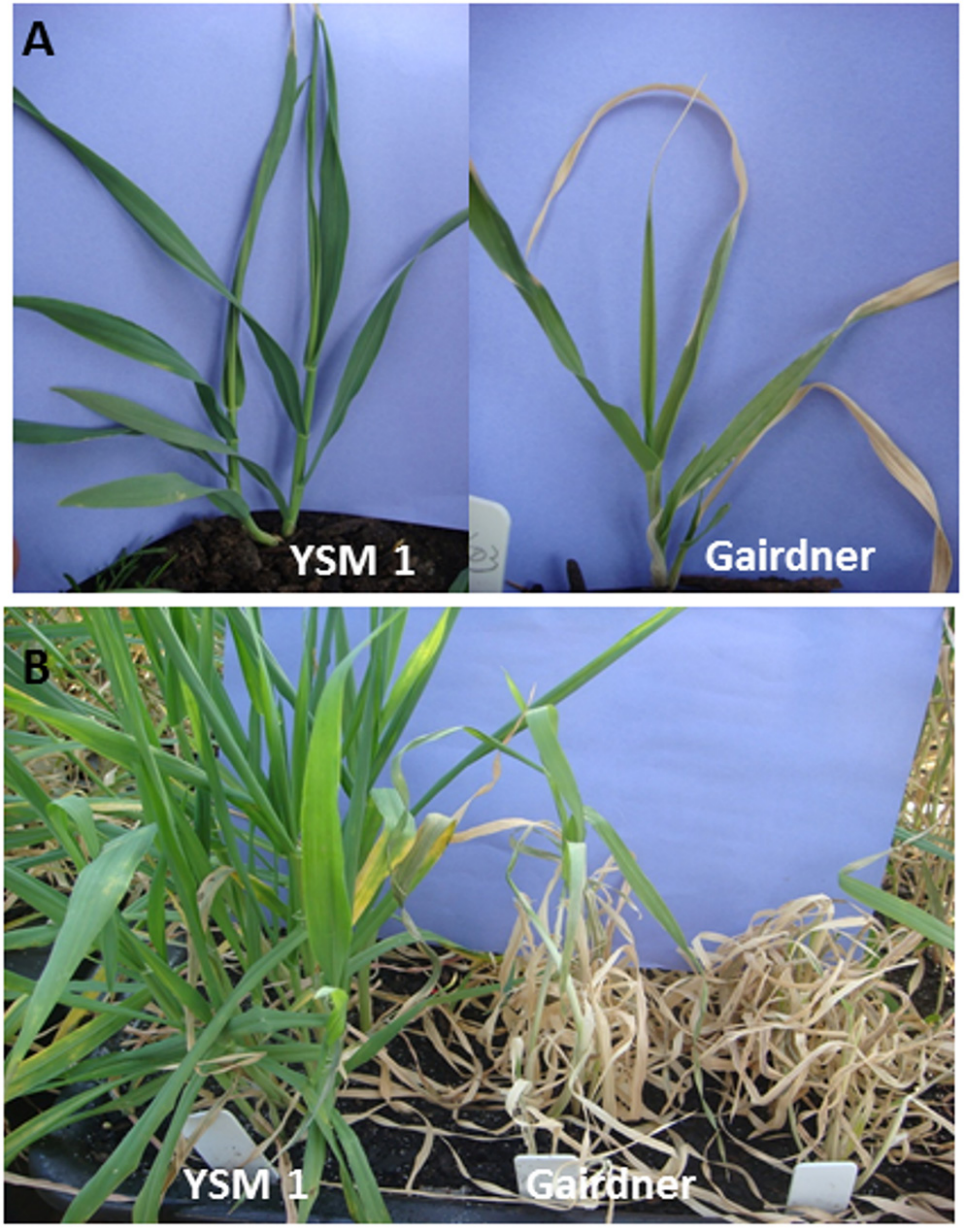
Comparison of salinity tolerance of two parental varieties YSM1 and Gairdner. (A) after 2-week SalinityD treatment and (B) after 4-week SalinityD treatment.

In both SalinityD and SalinityW treatments of the summer trial, TAM704227 showed the most tolerance with a damage score of only 2.0, while Naso Nijo and Gairdner were the most sensitive with scores of greater than 7.0. TX9425 showed very good tolerance to SalinityD treatment (with a score of 2.5 after two-weeks treatment in the summer trial and 3.5 after four-weeks treatment in winter trial) but became sensitive under SalinityW treatment (scored 6.0 after two week treatment in summer trials and 6.3 after three-week treatment in winter trials) (Table 1; please note that only scoring data for the summer trials are shown). Difference in leaf chlorosis under SalinityD stress (in summer trials) between the two parental varieties YSM1 and Gairdner could be detected after one-week treatment (Fig 1A) and became significant after four-week treatment (Fig 1B).

Leaf Na/K ratio was significantly higher (3–5 fold) in YSM1 than that in Gairdner under both SalinityD and SalinityW treatments. The leaf K^+^ contents in both two varieties were much lower under SalinityW stress than those under SalinityD stress. Similarly, the average leaf K^+^ content of DH lines under SalinityD stress (234.20±6.86) was 2-fold higher than that under SalinityW stress (110.39±2.58). The Na/K ratio in plants under SalinityW stress (5.24±0.11) was 2-fold higher than those treated with SalinityD stress (2.13±0.08). However, no significant difference was detected for the leaf Na^+^ content when comparing these two treatments (*p<0*.*05*) (Table 2).

**Table 2:** Comparison of leaf Na content, K content and Na/K ratio in 122 DH lines and two parents under SalinityD and SalinityW treatments.

Genotypes		SalinityD			SalinityW	
	Na^+^ content (μM)	K^+^ content (μM)	Na/K ratio	Na^+^ content (μM)	K^+^ content (μM)	Na/K ratio
**Gairdner**	435.03±28.93^a^	207.92±46.92	2.20±0.66	742.43±53.66	106.83±5.56	7.02±1.37
**YSM1**	308.68±15.14	351.09±17.81	0.81±0.07	573.23±47.24	124.93±5.53	4.58±0.18
**DH lines**	468.77±19.93	234.20±6.86	2.13±0.08	551.66±12.66	110.39±2.58	5.24±0.11

^a^ Data are mean±SE.

**Fig 2:**
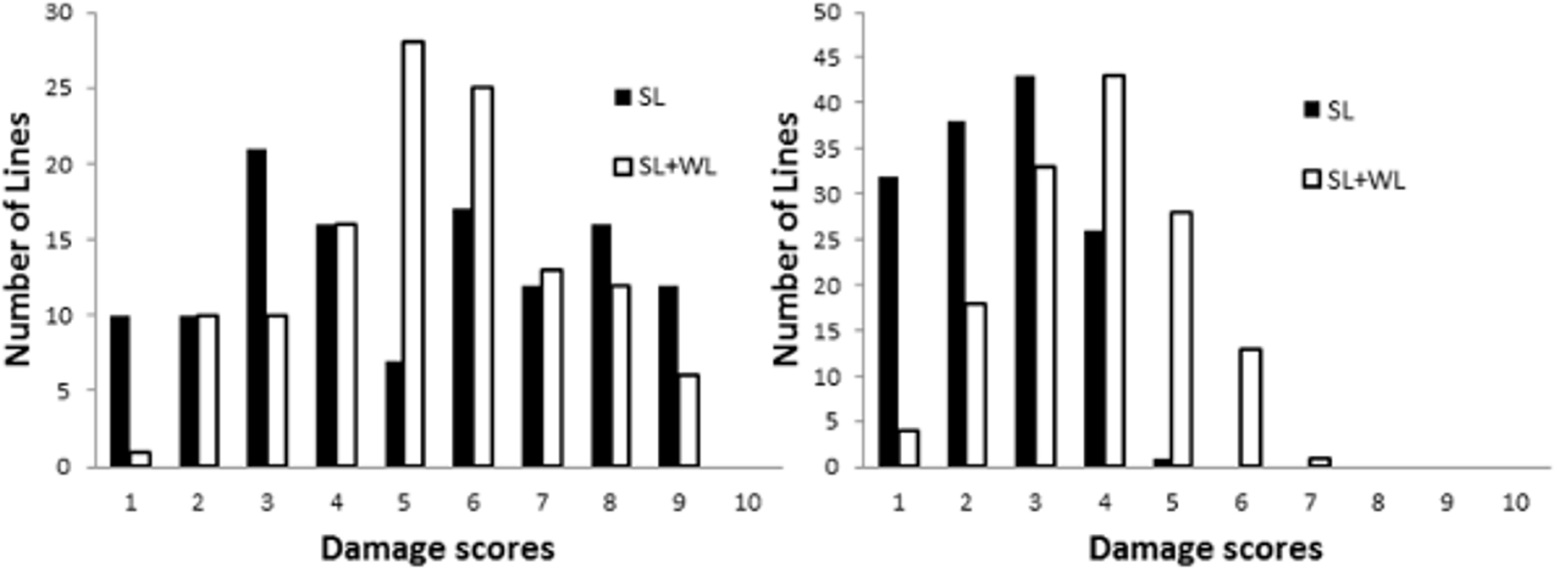
Distribution of salinity tolerance (damage scores) of DH lines under drained and waterlogged conditions in summer trials (A) and winter trials (B) (parental varieties included). Solid bars: the number of individuals after four-week SalinityD treatment and hollow bars: the number of individuals after two-week SalinityW treatment. ‘Y’ = YSM1, ‘G’ = Gairdner.

DH lines showed significant differences in salinity tolerance in both winter and summer trials under drained and waterlogged conditions (*p*<0.01) (Table 3). Frequency distributions for plant damage scores were all approximately normally distributed (Fig 2A and 2B). Significant differences in Na/K ratio also existed among the DH lines under both SalinityD and SalnityW treatments (*p*<0.01) (Table 3).

**Table 3:** ANOVA results of 122 DH lines in salinity tolerance under various environments and leaf Na/K ratio in summer and winter trials (*p*<0.01).

Source Of Variation	Summer trials								Winter trials			
	Damage scores Under SalinityD		Damage scores under SalinityW		Leaf Na/K ratio Under SalinityD		Leaf Na/K ratio Under SalinityW		Damage scores under SalinityD		Damage scores under SalinityW	
	MS	F value	MS	F value	MS	F value	MS	F value	MS	F value	MS	F value
**Genotype**	12.22	7.84**	7.35	3.79**	1.66	1.61**	3.29	1.91**	2.31	1.75**	3.12	2.70**
**Replication**	12.96	8.31**	42.15	21.77**	0.36	0.35	20.29	11.78**	0.18	0.13	1.73	1.50
**Error**	1.56		1.93		1.03		1.72		1.32		1.15	

** significant at the 1% level

* significant at the 5% level.

### Evaluation of plant development in summer trials

**Fig 3:**
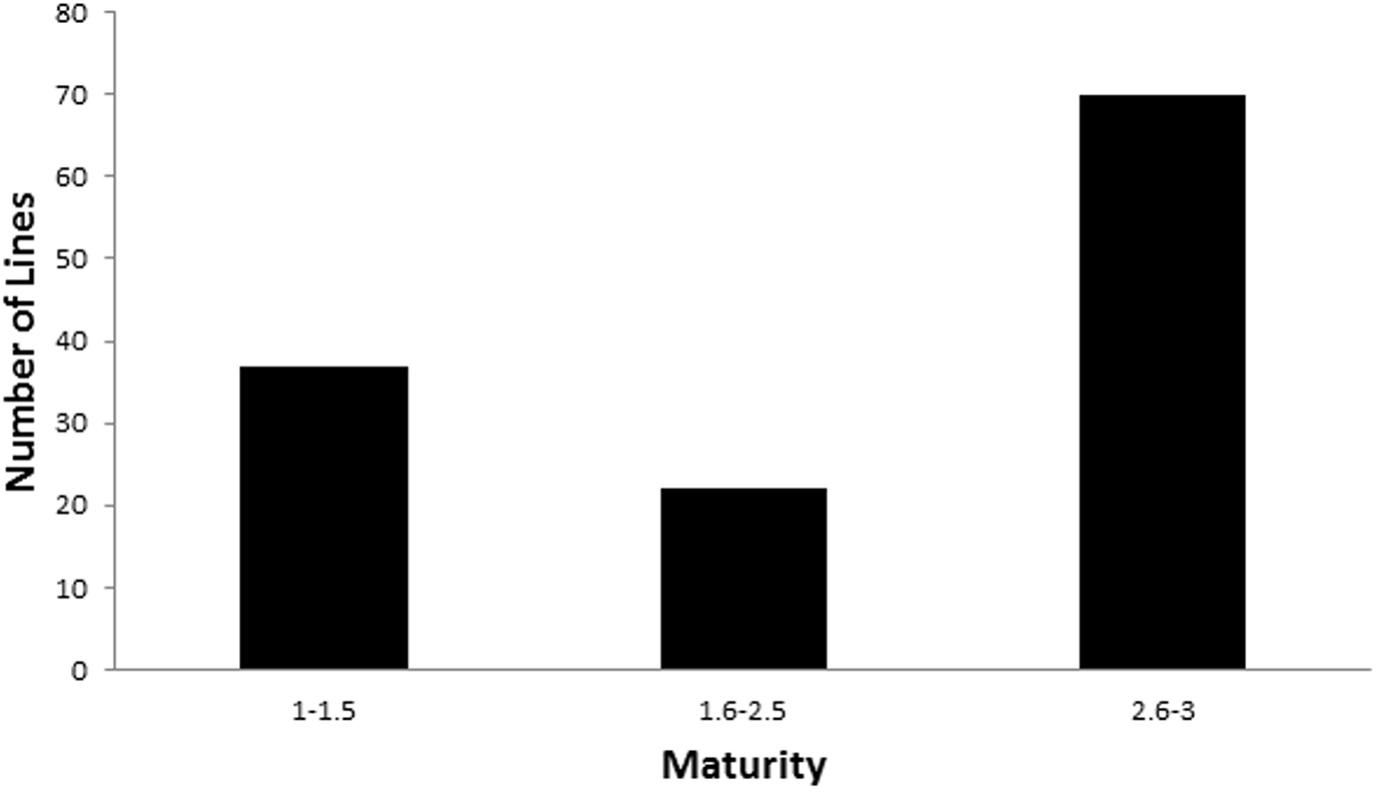
Distribution of plant development scores of DH lines after 3-week SalinityD treatment in the summer trial (parental varieties included).

Plant development stage was scored only in the summer trial as the effect of vernalisation genes cannot be clearly detected in winter time. The DH lines were scored as 1 (plants that stayed at tillering stage) to 3 (plants that reached heading stage). The average scores of two parental varieties are 2.25 for YSM1 (a Zadoks score of 45) and 3 for Gairdner (a Zadoks score of 65), respectively (Table 1). The distribution for flowering time of DH lines is shown in Fig 3. There were 48 lines staying at tillering or later than YSM1, while 74 lines were earlier than YSM1. The frequency distribution skewed towards the non-vernalisation-required parent Gairdner (Fig 3). Among the 48 late-flowering lines, 29 lines required strict vernalisation conditions and remained at tillering stage (Fig 3). The salinity tolerance was also compared between three groups of DH lines. In the summer trial, DH lines with flowering time score ranging from 2.6 to 3 were significantly less tolerant to SalinityW stress than lines at tillering stage (1–1.5) (*p*<0.05). For SalinityD treatment, the significant difference between lines at tillering stage (1–1.5) and those at booting stage (1.6–2.5) or heading stage (2.6–3) was only observed after four-week treatment (Table 4) (*p*<0.05).

**Table 4:** Comparison of SalinityD and SalinityW tolerance (damage scores) in two groups of DH lines differing in flowering time (summer trials).

Score ranges of flowering time	2-week treatment		3-week treatment	
	SalinityD	SalinityW	SalinityD	SalinityW
**1–1.5**	3.08	2.88	4.30	4.19
**1.6–2.5**	2.16	4.05	4.78	5.69
**2.6–3**	2.91	4.38	5.30	6.37

### QTL conferring salinity tolerance and leaf Na/K ratio

**Fig 4:**
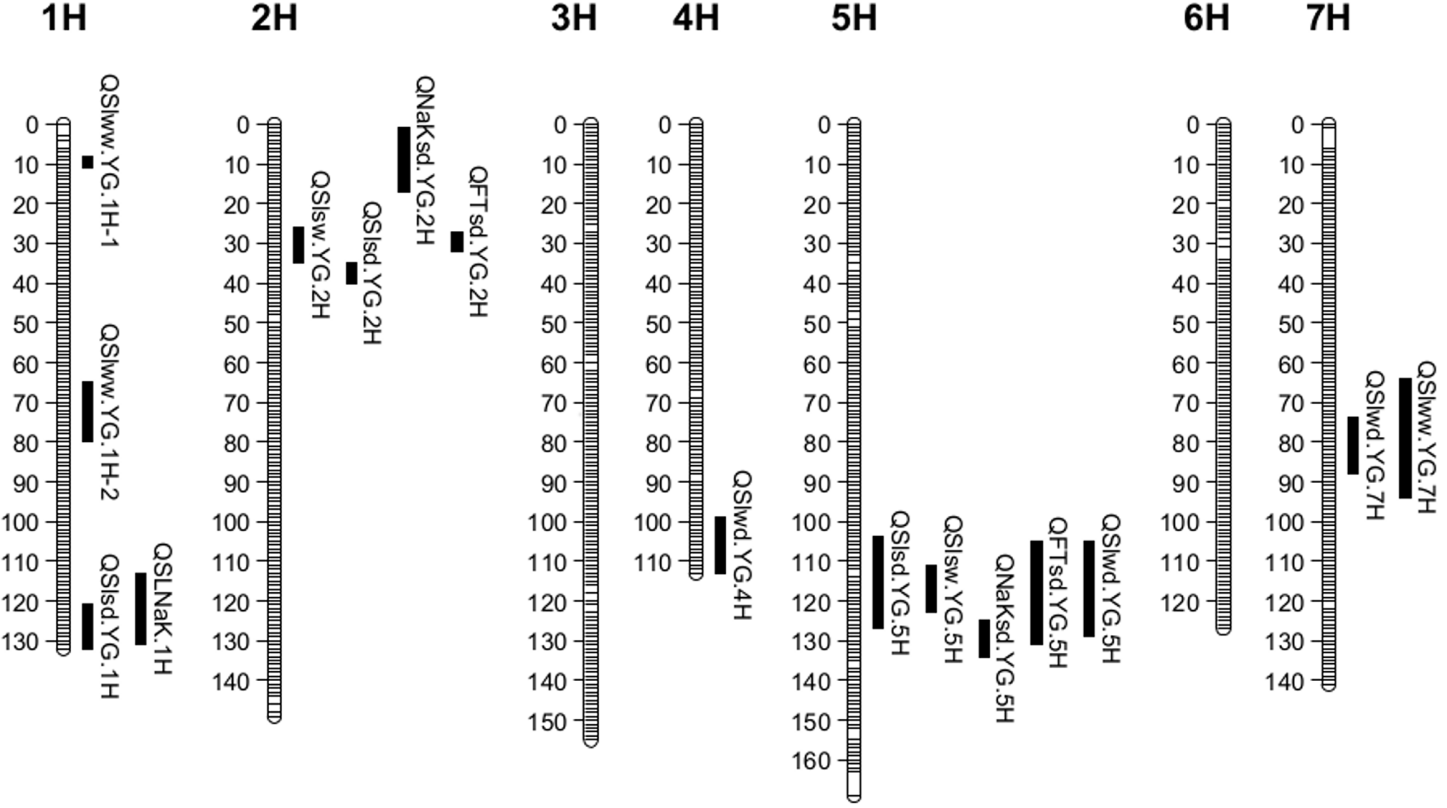
QTL associated with salinity tolerance, flowering time and leaf Na/K ratio. Map distances in centiMorgan (cM) are on the left.

In the summer trial, three QTL were identified for SalinityD tolerance (Table 5). They are located on the long arm of chromosome 1H, the short arm of chromosome 2H and the long arm of chromosome 5H, respectively (Fig 4). These three QTL are designed as *QSlsd*.*YG*.*1H*, *QSlsd*.*YG*.*2H* and *QSlsd*.*YG*.*5H*, where *Sl* represents for salinity and *sd* for SalinityD treatment in summer trials. These QTL explained a total of nearly 45% of phenotypic variation (Table 5). Only two significant QTL, *QSlsw*.*YG*.*2H* and *QSlsw*.*YG*.*5H*, with sw representing for SalinityW treatment in summer trials, were identified for SalinityW tolerance. These two QTL are located at similar positions to these identified under SalinityD treatment (Fig 4; Table 5), while the QTL on 1H identified for SalinityD tolerance was not found in SalinityW treatment. For the trait of leaf Na/K ratio, three QTL, *QNaKsd*.*YG*.*1H*, *QNaKsd*.*YG*.*2H* and *QNaKsd*.*YG*.*5H*, were identified for SalinityD tolerance (Fig 4). The major QTL *QNaKsd*.*YG*.*5H* was located at the position of 130.9 cM on chromosome 5H with LOD value 5.62, explaining 16.0% of the phenotypic variation (Table 5). No significant QTL was detected for Na/K ratio under SalinityW treatment.

**Table 5:** QTL for SalinityD tolerance (SD), SalinityW tolerance (SW), flowering time (FT) and leaf Na/K ratio (SDNaK) identified in the DH population of Gairdner × YSM1 in summer trials (Only QTL with LOD value>3.0 were shown).

Traits	QTL	Linkage Group	Position (cM)	Nearest marker	LOD	R^2^ (%)
**SD**	*QSlsd*.*YG*.*1H*	1H	129.8	3665819S^a^	7.06	16.0
	*QSlsd*.*YG*.*2H*	2H	39.5	3260104S	7.78	17.9
	*QSlsd*.*YG*.*5H*	5H	125.5	3258566S	4.40	9.3
**SW**	*QSlsw*.*YG*.*2H*	2H	31.5	3265675D^b^	3.70	10.2
	*QSlsw*.*YG*.*5H*	5H	120.9	3398786S	6.31	18.4
**SDNaK**	*QNaKsd*.*1H*	1H	117.6	3398249D	3.35	9.1
	*QNaKsd*.*2H*	2H	8.6	3255845S	3.64	9.9
	*QNaKsd*.*5H*	5H	130.9	3273502D	5.62	16.0
**FT**	*QFTsd*.*YG*.*2H*	2H	31.5	3265675D	15.21	38.0
	*QFTsd*.*YG*.*5H*	5H	111.7	3398891S	3.89	7.7
**FT adjusted SD**	*QSlsd*.*YG*.*1H-1*	1H	21.7	3259735D	3.53	5.9
	*QSlsd*.*YG*.*1H-2*	1H	129.8	3256276D	10.62	20.4
	*QSlsd*.*YG*.*2H*	2H	39.5	3260104S	4.43	7.5
**FT adjusted SW**	*QSlsw*.*YG*.*5H*	5H	120.9	3398786S	3.45	8.8
**SDNak adjusted SD**	*QSlsd*.*YG*.*1H-1*	1H	21.7	3259735D	2.97	5.8
	*QSlsd*.*YG*.*1H-2*	1H	131.1	3429901D	5.97	13.8
	*QSlsd*.*YG*.*2H*	2H	31.3	3256717S	9.34	23.2

^a ‘^S’ stands for SNP markers

^b ‘^D’ stands for DArT markers.

In the winter trial, four QTL for SalinityD tolerance, *QSlwd*.*YG*.*3H*, *QSlwd*.*YG*.*4H*, *QSlwd*.*YG*.*5H* and *QSlwd*.*YG*.*7H* were identified under drained conditions, with *wd* representing SalinityD treatment in winter trials (Fig 4). These four QTL could explain a total of 38% of phenotypic variation (Table 6). The major one *QSlwd*.*YG*.*7H* is located at the position of 80.4 cM on chromosome 7H with a LOD value of 7.09 (Fig 4; Table 6). A total of three QTL, *QSlww*.*YG*.*1H-1*, *QSlww*.*YG*.*1H* -2 and *QSlww*.*YG*.*7H* (ww representing SalinityW treatment in winter trials), were detected, accounting for 38.2% of phenotypic variation (Table 6).

**Table 6:** QTL for SalinityD tolerance (SD) and SalinityW (SW) identified in the DH population of Gairdner × YSM1 in winter trials (Only QTL with LOD value>3.0 were shown).

Traits	QTL	Linkage Group	Position (cM)	Nearest marker	LOD	R^2^ (%)
**SD**	*QSlwd*.*YG*.*4H*	4H	103.7	3271960D	3.79	8.1
	*QSlwd*.*YG*.*5H*	5H	120.9	3398786S	4.04	8.7
	*QSlwd*.*YG*.*7H*	7H	80.4	3432693D	7.09	16.1
**SW**	*QSlww*.*YG*.*1H-1*	1H	10.3	3429856D	5.99	15.4
	*QSlww*.*YG*.*1H-2*	1H	72.2	3666342S	4.66	11.5
	*QSlww*.*YG*.*7H*	7H	82.3	3262160S	6.35	13.3

### QTL associated with plant development stages (flowering time) in summer trials

Two QTL, *QFTsd*.*YG*.*2H* and *QFTsd*.*YG*.*5H*, were identified for plant development, with FT representing for flowering time. The QTL on chromosome 2H (position 31.5 cM) explained 38.0% of phenotypic variation, which shared the same closest maker as *QSlsw*.*YG*.*2H* (31.5.00 cM) and is 8 cM away from *QSlsd*.*YG*.*2H* (Fig 4). *QFTsd*.*YG*.*5H* with a LOD value of 3.89, was mapped at a similar position as *QSlsd*.*YG*.*5H and QSlsw*.*YG*.*5H* (Fig 4; Table 5).

### Effects of flowering time on salinity tolerance

**Fig 5:**
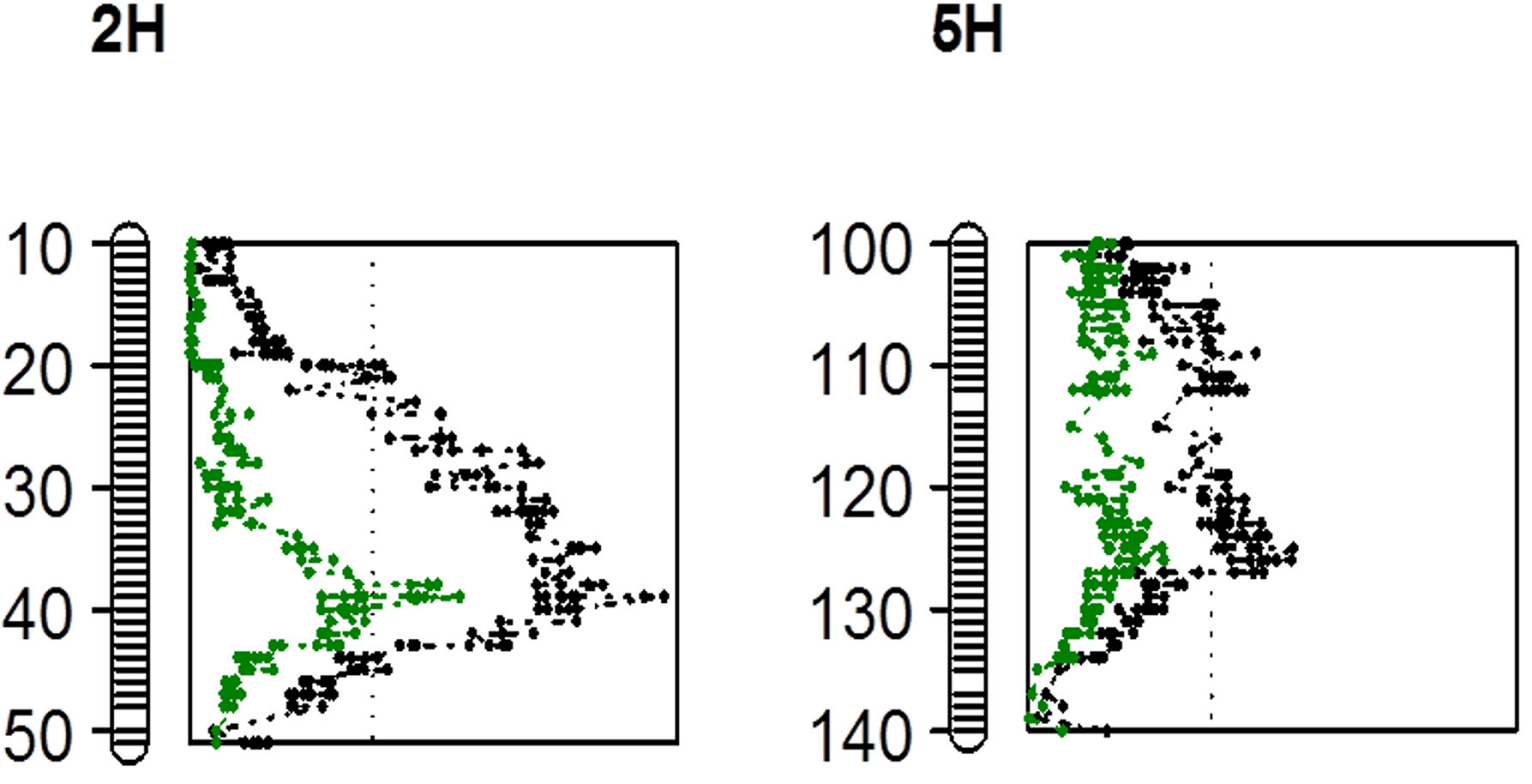
Comparison of LOD values of QTL associated with SalinityD tolerance on chromosome 2H (A) and 5H (B) before and after flowering time was used as a covariate. Map distances in centiMorgan (cM) shown to the left. The vertical dotted line indicates the significance threshold of LOD 3.0. Black: LOD value range of original QTL; Green: new LOD value range of QTL when trait of flowering time was used as a covariate.

DH Lines with late flowering lines showed greater tolerance than lines with early flowering (Table 4). QTL associated with plant development genes identified in this study showed significant effects on salinity tolerance in summer trials. The positions of the QTL associated with flowering time (*QFTsd*.*YG*.*2H* and *QFTsd*.*YG*.*5H*) were found to be overlapped with those for salinity tolerance. To quantify the effects of plant development stages on SalinityD and SalinityW tolerance, flowering time scores were used as a covariate when analysing QTL for salinity tolerance in summer trials. The LOD value of *QSlsd*.*YG*.*2H* was significantly reduced from 7.78 to 4.43, with R^2^ decreasing from 17.9% to 7.5% when plant development stage was considered (Fig 5A, Table 5). The QTL *QSlsw*.*YG*.*5H* for SalinityW tolerance, became insignificant (LOD<3.0) when flowering time was used as a covariate. Similarly, when flowering time was accounted for, the LOD value of *QSlsw*.*YG*.*5H* decreased from 6.31 to 3.45, while *QSlsd*.*YG*.*5H* became insignificant (LOD<3.0) (Fig 5B, Table 5). Meanwhile, QTL mapped on chromosome 1H (position 129.8 cM) remained highly significant with an even increased LOD value and its position was not affected by flowering time (Table 5). Moreover, a new QTL on 1H, *QSLsd*.*YG*.*1H-1* with the closest marker being located at 21.7 cM was detected when using flowering time as the covariate (Table 5). Further studies may be needed to find the relationship between flowering time and these two QTL on 1H.

## Discussion

### QTL for salinity varied under different environments

Many QTL were reported for salinity tolerance based on various traits, for example, germination rates, chlorophyll content, chlorophyll florescence (Fm/Fv), tissue proline and carbohydrate content, relative water content, coleoptile and radicle length, wet and dry weights of tissues and shoot sodium content [2,11–14]. A QTL for Na^+^ accumulation was mapped on 1HL using 150 DH lines derived from the cross of Clipper×Sahara 3771 [15,42]. The locus which is closely linked with the clustered markers, ABC257, cMWG733, BCD808a, and CDO669b, was named as *HvNax4* and explained 79% of the variation in the trait [15]. In another DH population from Harrington×TR306 cross, a single QTL for salinity tolerance at germination stage was also mapped on 1HL with the nearest marker of ABC261. This QTL was located on a similar position to *HvNax4* [15,43]. By comparing the above five markers closely linked to *HvNax4* in a barley consensus map [44] and locating them into a barley physical map, the QTL for salinity tolerance (*QSlsd*.*YG*.*1H*) identified in this study is located approximately 20 cM away from the position of *HvNax4*. Further QTL analysis for salinity tolerance using Na/K ratio as a covariate indicated that Na/K ratio showed no significant effects on the QTL mapped on chromosome 1H (Table 5). It should be commented though that the whole leaf Na and K analysis fails to account for intracellular sequestration of this ions and, therefore, may not necessarily be causally associated with salinity stress tolerance. This may also indicate that the QTL on chromosome 1H identified in this study is different from *HvNax4*. The two QTL identified on chromosome 1H in the winter trials under waterlogged condition were not found in either the summer or winter trials under drained conditions and no major QTL in these regions were reported before.

Two QTL *QSlsd*.*YG*.*2H* and *QSlsw*.*YG*.*2H* on chromosome 2H were identified in both drained and waterlogged trials in summer, explaining 17.9% and 10.2% of phenotypic variation, respectively. The position of those QTL is more than 20 cM away from a major QTL on chromosome 2H for salinity tolerance reported by [2]. However, *QNaKsd*.*YG*.*2H* leaf Na/K ratio identified from the summer trial is very close to that QTL [2]. In winter trials, no QTL located on chromosome 2H was identified. Instead, a novel QTL for salinity tolerance mapped on chromosome 4H was identified in the winter trial under drained conditions. QTL mapped on chromosome 5H with map interval 120.9–125.5 cM was identified in three of four different environments (salinity treatment in both summer and winter experiments and salinity + waterlogging treatment in summer experiments). The position of this QTL is different from that reported by Zhou *et al*. [14] but similar to those reported by Siahsar and Aminfar [45] who mapped a few physiological traits in this region.

*QSlwd*.*YG*.*7H* and *QSlww*.*YG*.*7H* identified in the winter trial were located into the same map interval as another Na^+^ accumulation QTL, named as *HvNax3*, which is located close to the middle of chromosome 7H and flanked by DArT marker bPb-1209 and microsatellite marker GBM1519 [16]. Fan *et al*. [46] also reported a QTL for salinity tolerance in a similar position on chromosome 7H. Those two QTL were also found to be located closely to a QTL identified by Zhou *et al*. [14] by comparison in the barley consensus map [44] and barley physical map constructed in this study.

For other QTL identified in the winter trial, *QSlww*.*YG*.*1H-2* was found to be mapped closely to a QTL associated with Na/K ratio reported before [47]. We also found *QSlww*.*YG*.*1H-2* was mapped into the same map interval with two QTL for leaf chlorosis and yellowing under two-week and four-week waterlogging respectively, although those two QTL only account for 7.1% and 5% of the phenotypic variation [48].

### Flowering time showed significant effects on plant tolerance to salinity

Two QTL were identified for plant development (trait of flowering time in this study) on 2HL and 5HL, based on plant development stage in the summer trial. Among them, *QFTS*.*YG*.*2H* was a major QTL with LOD value of 14.37 and explaining 36.4% of the phenotypic variation. This QTL is at the same position as previously reported photoperiod response locus (*PPD-H1*) [49–53]. The minor QTL on chromosome 5H was mapped to the same region as the major QTL for vernalisation requirements found in the YYXT × Franklin DH population [54], which is near *VRN-H1* by comparing the marker BCD265b [55] using a barley consensus map [44].

Later flowering or non-flowering DH lines showed generally better tolerance in summer trials (Table 4). When using plant development stage as a covariate to analyse QTL for salinity tolerance, the most significant reduction in QTL effects was observed for salinity tolerance QTL on both chromosome 2H and 5H. The LOD value of the QTL on chromosome 2H decreased from 7.78 to 4.43 under drained conditions and from 3.7 to insignificant under waterlogged conditions. Similarly, the LOD value of the QTL on chromosome 5H decreased from 4.4 to insignificant (<3.0) under drained condition and from 6.31 to 3.45 under waterlogged condition. The effect of development genes on stress tolerance has also been reported in many other studies [27,28]. A zinc finger transcription factor, BBX24 from *Chrysanthemum morifolium* has been identified to be associated with both flowering time and stress tolerance [56]. NAC (NAM, ATAF1,2 and CUC2) protein AtRD26 /ANAC072 from *Arabidopsis* and BnNAC485 from *B*. *napus* play roles in plant flowering time and ABA-mediated pathway in response to abiotic stresses [30]. At epigenetic level, KINASE BINDING PROTEIN1 (SKB1) can suppress *FLC* (Flowering Locus C) expression as well as a number of stress-responsive genes, including salt tolerance related genes, by binding to chromatin and increasing the histone methylation level. As a result, the phenotypes present in this mutant exhibit salt hypersensitivity, late flowering, and growth retardation [28,57]. Apart from genetic evidence, the hormone study in *Arabidopsis* showed that jasmonate (JA) can delay flowering occurrence and enhance protection against biotic stress [31]. Methyl jasmonate (MeJA) has also been found to act as a vital cellular regulator that mediates diverse developmental processes and defence responses against biotic and abiotic stresses [58]. Meanwhile, growth and developmental processes of plants can vary in response to stress conditions. During periods of environmental stress, it is common that plant transit to reproductive development earlier than in optimal growth environments [27,28]. However, there were plants prone to apply the ‘quiescence’ strategy to conserve the energy without shoot or leaf elongation and use ATP economically when exposed to stresses, for example the low-oxygen quiescence strategy (LOQS) applied by plants under submergence stress [59,60]. The ‘quiescence’ strategy may account for our results that late flowering lines were relatively more tolerant than the early-flowering plants (Table 4).

In conclusion, several significant QTL (with LOD>3.0) for salinity tolerance were identified under different environments. Environments showed significant effects on QTL identified for salinity tolerance. The major QTL on chromosome 1H (130.9 cM) *QSlsd*.*YG*.*1H* for SalinityD tolerance in the summer trial and the major QTL on chromosome 1H (10.3 cM) *QSlww*.*YG*.*1H-1* for SalinityW tolerance in the winter trial were not reported before from other environments. The QTL mapped on chromosome 2H in summer trials were not detected in winter trials while the QTL on chromosome 4H and 7H were only identified in the winter trial. Plant development stage associated locus *QFTsd*.*YG*.*2H* and *QFTsd*.*YG*.*5H* were found to have significant effects on salinity tolerance, as late flowering lines showed better salinity tolerance, which has provided new clues for understanding the mechanisms for plant tolerance to salinity stress.
